# Structure of Bacterial Community with Resistance to Antibiotics in Aquatic Environments. A Systematic Review

**DOI:** 10.3390/ijerph18052348

**Published:** 2021-02-27

**Authors:** Ana María Sánchez-Baena, Luz Dary Caicedo-Bejarano, Mónica Chávez-Vivas

**Affiliations:** 1 Department of Natural Sciences, Exact and Statistics, Faculty of Basic Sciences, Campus Pampalinda, Universidad Santiago de Cali, Cali Calle 5 # 62-00, Colombia; ana.sanchez15@usc.edu.co; 2 Department of Biomedical Sciences, Faculty of Health, Campus Pampalinda, Universidad Santiago de Cali, Cali Calle 5 # 62-00, Colombia; monica.chavez02@usc.edu.co

**Keywords:** aquatic environment, antibiotic-resistant bacteria, antibiotic resistance genes, environmental ecology, gene structure, systematic review

## Abstract

Aquatic environments have been affected by the increase in bacterial resistant to antibiotics. The aim of this review is to describe the studies carried out in relation to the bacterial population structure and antibiotic resistance genes in natural and artificial water systems. We performed a systematic review based on the PRISMA guideline (preferred reporting items for systematic reviews and meta-analyzes). Articles were collected from scientific databases between January 2010 and December 2020. Sixty-eight papers meeting the inclusion criteria, i.e., “reporting the water bacterial community composition”, “resistance to antibiotics”, and “antibiotic resistance genes (ARG)”, were evaluated according to pre-defined validity criteria. The results indicate that the predominant phyla were *Firmicutes* and *Bacteroidetes* in natural and artificial water systems. Gram-negative bacteria of the family *Enterobacteraceae* with resistance to antibiotics are commonly reported in drinking water and in natural water systems. The ARGs mainly reported were those that confer resistance to β-lactam antibiotics, aminoglycosides, fluoroquinolones, macrolides and tetracycline. The high influence of anthropogenic activity in the environment is evidenced. The antibiotic resistance genes that are mainly reported in the urban areas of the world are those that confer resistance to the antibiotics that are most used in clinical practice, which constitutes a problem for human and animal health.

## 1. Introduction

Anthropogenic activity directly affects aquatic environments and alters microbial community composition. Resistant microorganisms and antimicrobial drugs are continuously discharged into water systems, favored by medical, veterinary, agricultural and industrial practices [1,2]. The most important sources of pollution are industrial and municipal discharges, constituting the main suppliers of sewage to aquatic environments [2,3,4,5,6,7]. In aquatic environments, antibiotics contribute to bacterial stress, exerting a selective pressure that generates resistant bacteria and environmental deterioration. In addition to causing a strong negative impact on the health of humans, animals and plants, they also alter the biogeochemical cycles in which bacteria are essential [3,4,5].

Bacteria become antibiotic-resistant by mutations or by the horizontal transfer of antibiotic resistance genes (ARGs) [4,5,6]. Experimental evidence has demonstrated that ARGs are generally located on mobile elements such as transposons, conjugative plasmids or integrons, and play an important role in genetic exchanges among environmental microbiota, especially in aquatic environments [4]. Therefore, water systems, such as rivers, streams, waste water effluents and lakes, are recognized as one of the reservoirs and transmission routes for the aggregation and dissemination of antibiotic-resistant bacteria, and the transport and transfer of the ARGs [4,5,6,8].

ARG pollution has been well documented in natural water systems, such as rivers [2], lakes [7] and seas [6], and it has been reported that drinking water and wastewater treatment plant (WWTP) processes are shown to be incapable of completely removing ARGs [7,9].

The current discharge of antibiotic-resistant bacteria, ARGs and mobile genetic elements, combined with the selection pressure by antibiotics, may lead to the selection of bacteria resistant to antibiotics and accelerate the emergence of new resistance [3,5]. Efforts to determine the structure and composition of the bacterial communities impacted by resistance to antibiotics in different water systems will allow us to address this problem with better strategies to control the spread of resistance to antibiotics.

This review summarizes selected studies related to the occurrence and relative abundance of antibiotic-resistant bacteria and ARGs, published from 2010 to 2020. A systematic review was made according to the PRISMA guidelines [10], with multiple approaches to identify the majority of publications that address the goal of describing the microbiota of the aquatic environment with resistance to antibiotics. Then, from each retrieved study, we extracted data and performed qualitative analyses on the relative abundance of antibiotic-resistant bacteria at the phylum level, types of ARGs, and the academic information associated with the studies, all of which are described and discussed in more detail.

## 2. Materials and Methods

### 2.1. Search Strategy and Selection Criteria

This systematic review was conducted according to the PRISMA guidelines (preferred reporting items for systematic reviews and meta-analyses) [10]. The reports of information on bacterial communities in water systems (artificial and natural) and the presence of genes with resistance to antibiotics were consulted in electronic databases. Pubmed, Science Direct, Scopus, Scielo, PLOS, Hinari, Redalyc, Dialnet, Taylor, ProQuest were systematically searched for studies reported in the last 10 years. Pubmed was searched with a strategy incorporating MeSH (medical subject headings), and free text was used extensively to search the appropriate articles from these databases using the following combinations of keywords: “microbial communities”, “Bacterial population structure”, “antibiotic-resistant bacteria”, “Drug Resistance”, “antibiotic resistance genes”, “resistant bacteria in the environment”, “water systems”, “sewage”, “Wastewater”, “Water Pollutants”, “River Pollution”, “aquatic environment”, “Fresh Water”, “Seawater”, “Surface Waters ”, “Drinking Water”, “aquaculture”.

### 2.2. Eligibility Criteria

Each article included in this review had to meet the following criteria: (1) the search strategy was restricted to the English language, and the papers must concern an original research study, published in a scientific journal; (2) they must have information that describes aquatic environment bacterial communities (in surface waters and sediments) and bacteria with resistance to antibiotics from natural water systems (rivers, lakes, lagoons, estuaries, sea) and artificial water systems (wastewater, drinking water, aquaculture, water treatment plants); (3) the studies must have been reported between January 2010 and December 2020. We excluded systematic reviews, editorials and policy statements.

### 2.3. Search for Articles

**Figure 1:**
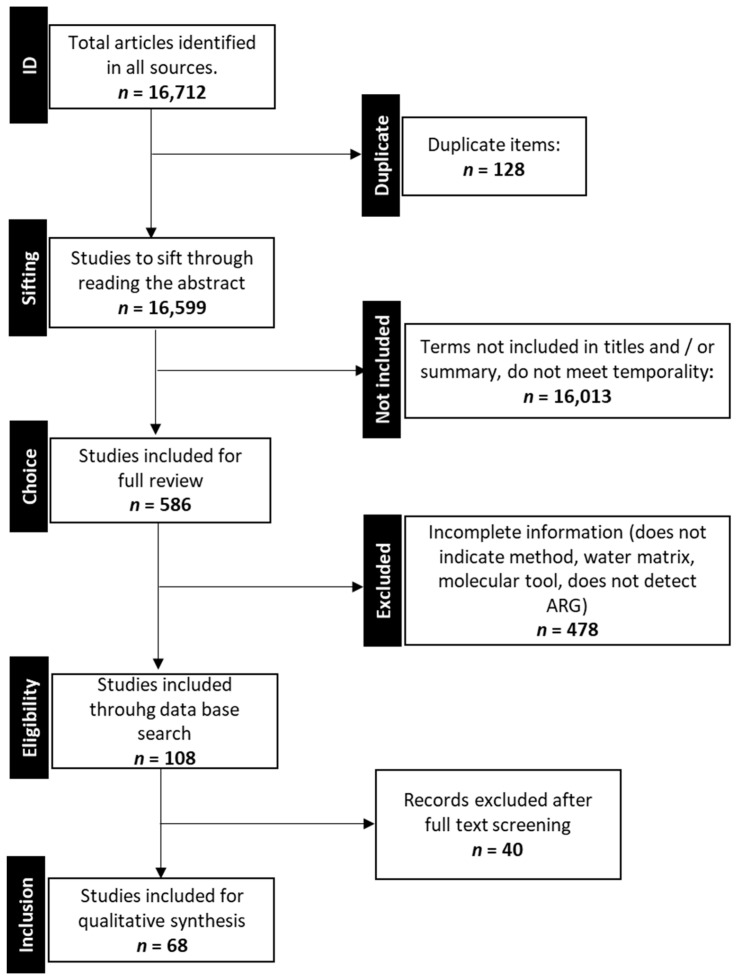
Flow diagram of search strategy and selection of articles reporting the structure of bacterial populations with resistance to antibiotics in aquatic environments.

With the articles initially identified with the search algorithm (Figure 1), the bibliographic information on the main author, publication date, journal, title and abstract were tabulated for each. Of these, the title and abstract were reviewed, and, according to the authors’ criteria, those that were relevant and would provide useful information for the review were selected, in order to subsequently review them in their entirety.

The articles identified in this first reading were read as a full text for the verification of inclusion and exclusion criteria.

### 2.4. Data Extraction

All articles included in the final analysis were reviewed by two authors independently using standardized data extraction tools prepared in the Microsoft Excel sheet to avoid bias and loss of information. The article selection was guided by the eligibility criteria previously stated. Disagreements between reviewers were resolved by reviewing the full article. When the two authors did not reach consensus over a specific article, an arbitrating reviewer was introduced. The PRISMA flow diagram (Figure 1) shows the number of articles in each step of the article selection process.

## 3. Results and Discussion

### 3.1. Search Results 

**Figure 2:**
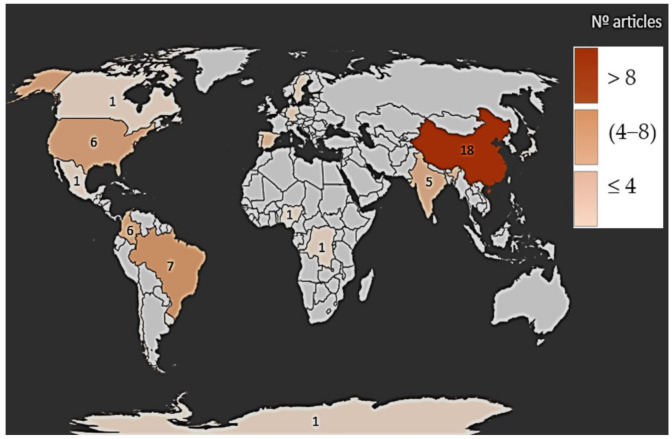
Worldwide aquatic environments studied. Number of articles per country: China (18), Chile (1), Colombia (6), India (5), Spain (4), USA (6), Brazil (7), Sweden (2), Portugal (2), Germany (2), South Korea (2), Vietnam (1), México (1), Jamaica (1), Uruguay (1), The Congo (1), Canada (1), Antarctica (1), Japan (1), Nigeria (1), Netherlands (1), multicentric (3).

We identified 16,727 potentially relevant studies through the database search. Of these, there were 128 duplicates, and 16,013 were excluded on the basis of title and abstract screening. A total of 68 studies [11,12,13,14,15,16,17,18,19,20,21,22,23,24,25,26,27,28,29,30,31,32,33,34,35,36,37,38,39,40,41,42,43,44,45,46,47,48,49,50,51,52,53,54,55,56,57,58,59,60,61,62,63,64,65,66,67,68,69,70,71,72,73,74,75,76,77,78] were included in the final review (Figure 1); they were conducted in the countries of Asia, Africa, Europe, North America, South America and Central America, as shown in the map in Figure 2 (Supplementary Materials: Table S1). Research was mainly conducted in Asia and Europe. The highest number of studies were from China (20.8% studies). Studies on natural waters (rivers, estuaries, sea, lakes and lagoons) were mostly conducted (considering ≥30 studies) in Asia (China, India, Tibet), Europe (Germany, Sweden, Spain), North America (USA, Canada), and South America (Brazil, Mexico, Colombia). As concerns studies in artificial waters (>19 studies), the study sites are distributed in Asia, Europe, and America, with the largest number of studies conducted in China.

### 3.2. Characteristics of Included Studies

**Figure 3:**
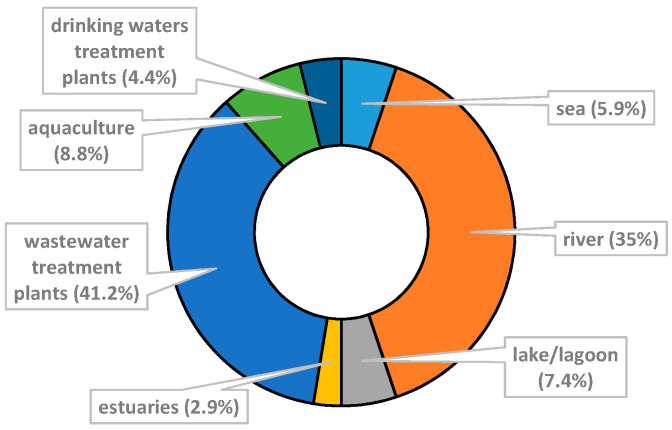
Proportions of studies (%) regarding population.

As illustrated in Figure 3, 41 articles analyzed the bacterial communities in natural waters. The greater proportion of samples collected were from rivers (35%; 31/86), and samples taken from lakes/lagoons (7.4%; 5/68), seas (5.9%; 4/68) and estuaries (2.9%; 2/68) represent a low percentage. The samples of artificial waters (37 articles) were taken mainly from wastewater treatment plants (WWTPs) (41.2%; 28/68), followed by drinking water treatment plant (4.4%; 4/68) and fish farms/ponds (8.8%; 6/68).

Metagenomics is the most widely used tool to study bacterial communities in aquatic systems, and represents 47.1% of the tools used. The most used method to characterize the bacterial communities was sequencing techniques, the Illumina technology was chosen 64.5% of the time, while the other 18.9% used Roche 454 pyrosequencing. The preferred variable region of the 16S rDNA gene sequenced to determine bacterial community composition was the V4 region (68.1%). Asian countries are the ones that use this technique the most (98%), and Latin American countries (5%) to a lesser extent.

Molecular tools such as pulsed field gel electrophoresis (PFGE) and multilocus typing sequence (MLST) are still used today to determine the composition of bacterial communities [43,44].

### 3.3. Structure of the Bacterial Community of the Aquatic Environment

The taxonomic classification of the bacterial population present in the microbial communities in the aquatic systems in the different studies reviewed is presented in Tables S2 and S3. Some of these investigations make a detailed classification, from the phylum to the dominant species. According to the articles reviewed, there is a greater abundance of α-Proteobacteria, β-Proteobacteria and γ-Proteobacteria in samples from natural and artificial waters.

**Figure 4:**
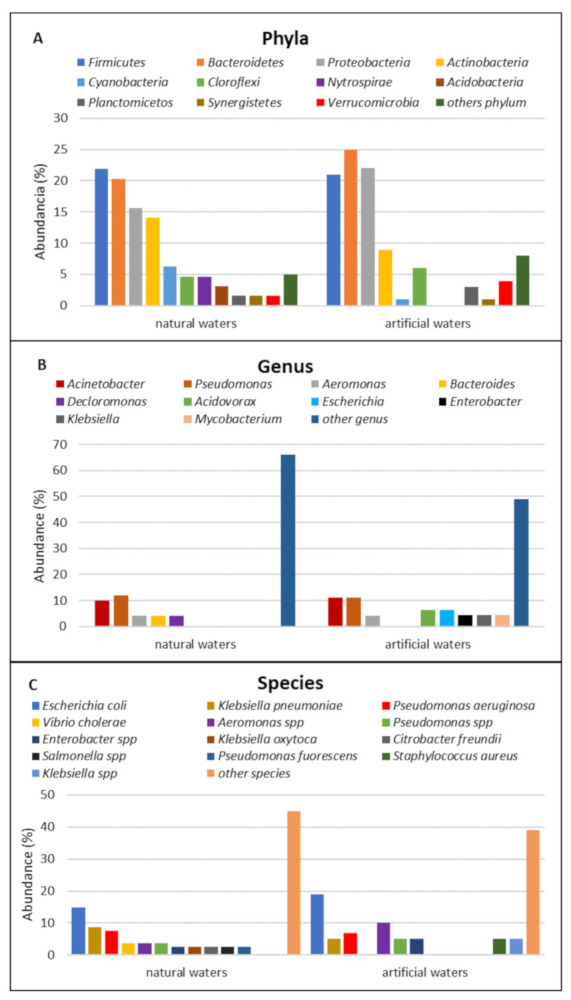
Taxa detected in the articles included in this systematic review. (**A**) Phyla, (**B**) Genus, (**C**) Species.

As seen in Figure 4A, natural waters presented a higher abundance of Firmicutes (22%) and Bacteroidetes (20%), while in artificial waters, Bacteroidetes (25%), Firmicutes (21%) and α-Proteobacateria (22%) were the most representative. The predominance of these phyla was to be expected since they are widely distributed in the environment, including in the intestinal tracts of humans and animals, and in soil, sediments and water [3,12,18,24,27,29].

The bacteria of the Enterobacteriaceae family were the most predominant in all aquatic environments [8,11,12,13,25,30,34,47,49,59,65,72], which shows the high impact that anthropogenic activity exerts on the environment.

The reports made at the species level show that Escherichia coli is the most abundant bacterium in natural and artificial waters (15% and 19%, respectively), followed by Pseudomonas aeruginosa (12% and 11%, respectively) and Acinetobacter spp. (10% and 11%, respectively) (Figure 4B). This shows the problem of the contamination of aquatic systems, which has been growing in proportion to industrial development and anthropogenic activity.

These bacteria are abundant in WWTPs in cities in Portugal, Spain, Germany, Sweden, South Korea, and Brazil [11,41,42,47,48,49,59]. However, they were also determined in treated waters and in natural water systems of marine environments, rivers and lagoons, mainly in Latin American countries [34,45,49,50,56,60].

These bacteria are pathogenic organisms that cause a large number of diseases, and are the primary cause of nosocomial infections, as stated by Decker et al. [1]. It is recommended to evaluate the procedures carried out in water treatment plants to try to optimize them and thus see a reduction in the potentially pathogenic bacterial loads of their effluents, before these are discharged into aquatic sources in areas of human and animal influence.

The analysis carried out on the microbiological composition of bacteria resistant to antibiotics in aquatic environments makes it possible to understand the population dynamics of microorganisms, and to quantify the risks to human health. However, the information on the biological source in the reviewed studies is not complete; some authors provide information in relation to the phylum, and not about the genera and species, or vice versa.

### 3.4. Abundance of ARGs in Aquatic Environments

In this systematic review, information was collected about the distribution of ARGs that are present in aquatic environments, and the frequency with which ARG selection and spread occurs in aquatic environments was determined.

The majority of ARGs reported are those that confer resistance to β-lactam antibiotics, aminoglycosides, fluoroquinolones and macrolides. However, of the studies, 12 (17.6%) did not have precise information on the types of ARGs found, as is the case for the reports made by Port et al. [27], Han et al. [48], Fang et al. [51], Pan et al. [54], Chen et al. [55], Eduardo-Correia et al. [57], Quintela-Baluja [42], Clarke et al. [61], Fernandes Cardoso de Oliveira et al. [62], Coutinho et al. [45], Miranda et al. [63], Lu et al. [64], Jiang et al. [65], and Qu et al. [66].

In Spain, they report the presence of genes for resistance to kanamycin, trimethoprim, erythomycin, vancomycin, streptomycin and aminoglycosides, β-lactams, efflux pumps and tetracycline, respectively. The study carried out by Lambirth et al. [58] reports the existence of genes for resistance to carbapenems and broad-spectrum β-lactamases. In accordance with the data provided around the world, the reports in Latin America, such as those made by Collins-Fairclough et al. [23], Delgado-Gardea et al. [34], Coutinho et al. [45] and Fresia et al. [56], present the same trend in the frequency of ARGs, with a predominance of resistance to β-lactams, aminoglycosides and macrolides.

**Figure 5:**
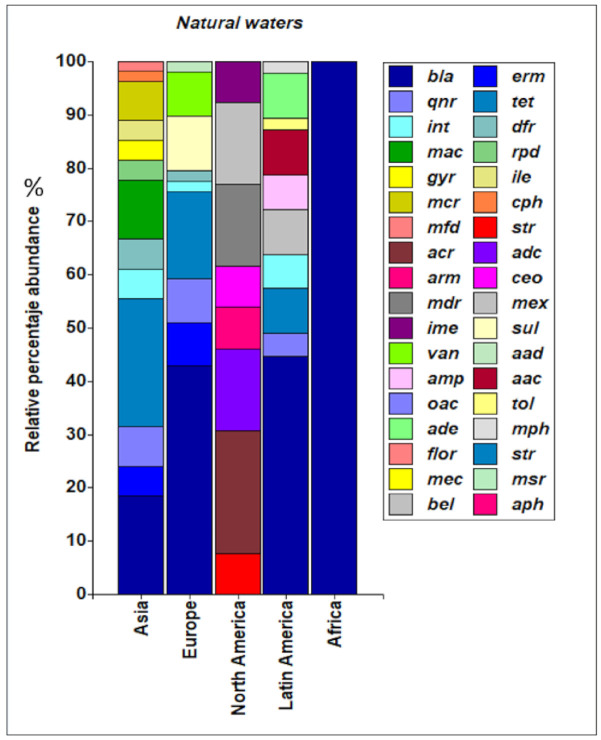
Types of genes reported in natural waters according to geographic distribution. *bla* (gene with resistance to betalactamase), *qnr*(gene with resistance to quinolones), *int* (integron), *mac* (gene with resistance to macrolides), *gyr* (gene encoding a subunit of DNA gyrase), *mcr* (gene with resistance to colistin), *mfd* (Mutation-Frequency-Decline: onfers resistance to the host nitrogen immune response), *acr (*gene that codes for acrosin), *arm* (Aminoglycoside resistance methyltransferase), *mdr* (The multiple drug resistance gene *mdr* encodes the so-called P-Glycoprotein), *ime* (Intron-mediated enhancement), *van (*gene with resistance to vancomicine), *amp* (gene with resistance to ampiciline), *oac* (gene with resistance to clarithromycin), *ade* (gene with resistance to tetracycline and glycylcycline), *floR* (gene with resistance to fluoroquinlones), *mec* (gene with resistance to methicillin), *bel* (type of extended spectrum beta-lactamase), *erm* (gene with resistance to erythromycin), *tet* (gene with resistance to tetracycline), *dfr* (gene with resistance to trimethoprim), *rpd* (gene with resistance to macrolides), *ile* (gene with resistance to quinolones), *cph* (gene with resistance to imipenem), *str* (gene with resistance to streptomycin), *adc* (is a type of A***mpC***, with resistance to ampicillin), *ceo* (gene with resistance to cefoperazone), *mex* (gene with resistance to tetracycline), *sul* (Sulfamethazine), *aad* (gene with resistance to aminoglycosides), *aac* (gene capable of acetylating fluoroquinolones), *tol* (gene with resistance to doxycycline), *mph (*gene with resistance to macrolides), *str* (gene with resistance to streptomycin), *msr* (gene with resistance to macrolides, lincosamides and streptogramins), *aph* (gene with resistance to aminoglycoside).

Concerning types of ARGs in natural water, there is a predominance of bla genes in Africa, Latin America and Europe, with 100%, 45% and 40%, respectively [11,13,14,15,16,21,22,24,25,29,46,47,49,50,59,67,68,69,78] (Figure 5). In Asian countries, tet genes are reported more frequently (≈15%) [14,16,18,70,71]. In North America, only one study reported the presence of acr genes (21%) [12].

These data corroborate the fact that the most widely used antibiotics in the world are those that reach natural water sources, allowing bacteria to adapt in these environments and increasing the resistome. For example, in China, there is a high production of tetracycline, and some of these studies were carried out with samples of water from rivers or natural water sources close to pharmaceutical companies, so it makes sense that the genes reported have been those that confer resistance to tetracyclines [14].

There is a low number of studies in relation to the types of ARG that circulate in the water systems of the countries of Africa; only one study was found on natural sources. This situation makes it difficult to gain clarity on the resistome worldwide [67].

**Figure 6:**
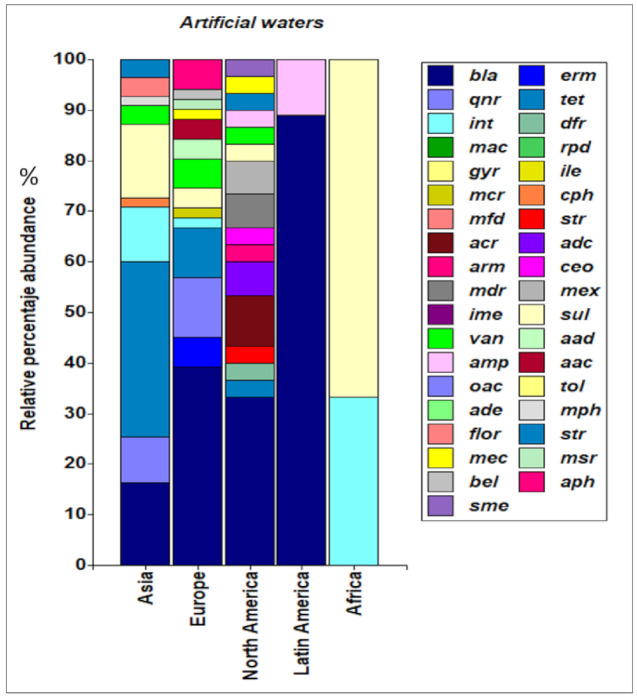
Types of genes reported in artificial waters according to geographic distribution. *bla* (gene with resistance to betalactamase), *qnr*(gene with resistance to quinolones), *int* (integron), *mac* (gene with resistance to macrolides), *gyr* (gene encoding a subunit of DNA gyrase), *mcr* (gene with resistance to colistin), *mfd* (Mutation-Frequency-Decline: onfers resistance to the host nitrogen immune response), *acr (*gene that codes for acrosin), *arm* (Aminoglycoside resistance methyltransferase), *mdr* (The multiple drug resistance gene *mdr* encodes the so-called P-Glycoprotein), *ime* (Intron-mediated enhancement), *van (*gene with resistance to vancomicine), *amp* (gene with resistance to ampiciline), *oac* (gene with resistance to clarithromycin), *ade* (gene with resistance to tetracycline and glycylcycline), *floR* (gene with resistance to fluoroquinlones), *mec* (gene with resistance to methicillin), *bel* (type of extended spectrum beta-lactamase), *sme (*gene with resistance to carbapenems), *erm* (gene with resistance to erythromycin), *tet* (gene with resistance to tetracycline), *dfr* (gene with resistance to trimethoprim), *rpd* (gene with resistance to macrolides), *ile* (gene with resistance to quinolones), *cph* (gene with resistance to imipenem), *str* (gene with resistance to streptomycin), *adc* (is a type of A***mpC***, with resistance to ampicillin), *ceo* (gene with resistance to cefoperazone), *mex* (gene with resistance to tetracycline), *sul* (Sulfamethazine), *aad* (gene with resistance to aminoglycosides), *aac* (gene capable of acetylating fluoroquinolones), *tol* (gene with resistance to doxycycline), *mph (*gene with resistance to macrolides), *str* (gene with resistance to streptomycin), *msr* (gene with resistance to macrolides, lincosamides and streptogramins), *aph* (gene with resistance to aminoglycoside).

Regarding the presence of the types of ARGs in artificial waters, the differences according to geographical location are notable. In Europe, North America and Latin America, the most prevalent ARGs are the bla genes, with approximate prevalence values of 40%, 34% and 89%, respectively [11,15,38,40,44,47,59,60,72,76]. In Africa, the sul genes (67%) [41], and in Asia the tet genes, are the most reported (30%) [32,35,48,65,70,71] (Figure 6). Most of these genes are reported in WWTPs, which indicates that the processes carried out there must be optimized to reduce the load of ARGs that reaches these plants, especially from products generated from clinical and domestic activities.

**Figure 7:**
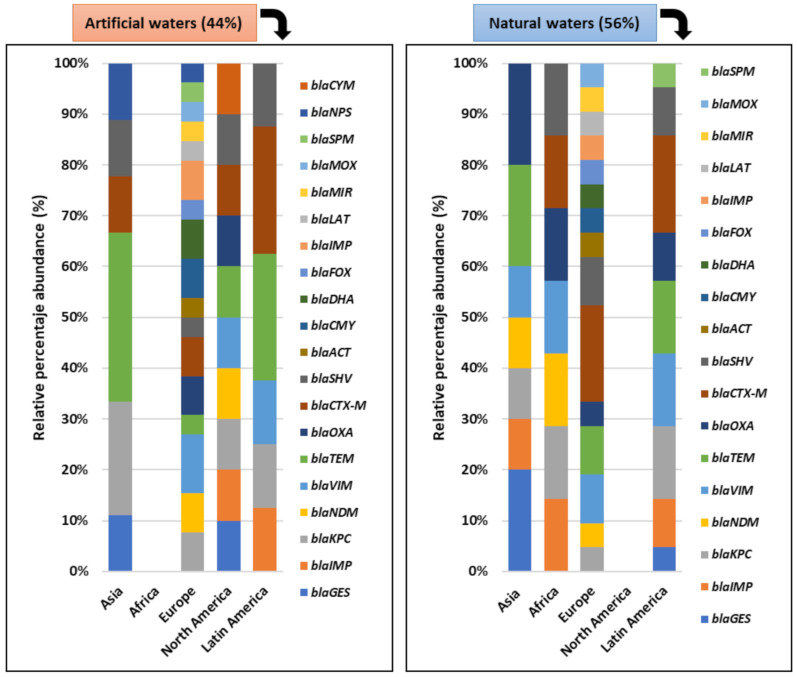
Abundance of bla genes in natural and artificial waters. *blaCYM* (substrate specificity for cephalosporins), *bla*NPS (have partial hydrolyzing abilities against penicillins and cephalosporin), *bla*SPM confers resistance to carbapenem), *bla*MOX (plasmid-mediated AmpC-type beta-lactamases), *bla*MIR (confer resistance to oxyimino- and alpha-methoxy beta-lactams), *bla*LAT (confer resistance to cephamycin), *bla*IMP (confer resistance to carbapenem, cephalosporin, cephamycin, penam, penem), *bla*FOX (conferred resistance to broad-spectrum cephalosporins and cephamycins), *bla*DHA (confer resistance to cephamycin and cephalosporin), *bla*CMY (confer resistance to cephamycin), *bla*ACT (confer resistance to actinomycin), *bla*SHV (confer resistance to carbapenem), *bla*CTX (confer resistance to cefotaxime acid), *bla*OXA (confer resistance to oxazocillin), *bla*TEM (confer resistance to termocilline), *bla*VIM (confer resistance to cephamycin), *bla*NDM (confer resistance to penem), *bla*KPC (confer resistance to monobactam, carbapenem, cephalosporin, penam), *bla*IMP (confer resistance to penam), *bla*GES (confer resistance to penam).

A high frequency of bla genes is reported in the aquatic environment (Figure 7). In natural water systems, the bla_TEM_, bla_GES_, and bla_OXA_ genes have a frequency greater than 20% in Asian countries [16,19,78]. The bla_CTX_ (20%), bla_TEM_ (10%) and bla_VIM_ (10%) genes are more common in Europe [11,13,14,22,24,28,29,47], while bla_CTX_ (19%), bla_TEM_ (14%), bla_KPC_ (14%) and bla_VIM_ (14%) genes are more common in Latin America [15,25,46,49,50,59]. Laffite et al. [67] reported an abundance of bla_CTX_, bla_IMP_, bla_KPC_, bla_NDM_, bla_OXA_, bla_SHV_, and bla_VIM_ genes (14% each) in the rivers of Africa.

In relation to the reports of bla genes in artificial waters, in North America, bla_CTX_, bla_GES_, bla_NDM_, bla_VIM_, bla_SHV_, bla_OXA_, bla_MOX_, bla_KPC_, bla_IMP_ and bla_TEM_ genes were identified in WWTP effluents [76], and in Europe, the bla_VIM_ gene (12%) was the most prevalent [11,48]. In Asia, a higher abundance of bla_TEM_ (33%) was detected, followed by bla_KPC_ (22%) in water samples from WWTP and aquaculture [36,38,73], while in Latin American countries, bla_CTX_ (25%) and bla_TEM_ (25%) were the most prevalent [15,43,44,61]. No studies were found reporting on these genes in artificial waters from Africa.

Several authors have determined the stability of ARGs in aquatic systems, according to seasonal changes. Alexander et al. [47] found that the abundance of ARGs in the receiving streams of a WWTP follows a pattern of temporal appearance.

Mittal et al. [77] found seasonal variations in the Yamuna river, New Delhi. Before the monsoon, the most abundant ARGs are those that code for efflux pumps (and *MtrC-MtrD-MtrE*), and after the monsoon, the most abundant are those that confer resistance to fluoroquinolones. The genes that confer resistance to rifampin, macrolides, chloramphenicol, tetracycline, phenicol, aminocoumarin, β-lactams, lipopeptides, elfamycin, polymyxins, aminoglycosides, isoniazid, trimethoprim, and lincosamide were found in the river all the time. In accordance with these reports, in the Lahn river, which is heavily impacted by agricultural activities, an increase in *bla_CTX-M_* concentrations was demonstrated after the rains [22].

Seasonal changes have also been evaluated in the dynamics of the structures of the communities of the aquatic environments. In Mexico, the analysis carried out in the Basaseachi waterfall and its main rivers found a higher count of total coliforms in autumn, while in spring the count was lower [34]. The presence of resistant bacteria and various ARGs (*bla*_GES_*, Int-1, adeS, acrA, acrB, tolC, mex B, mex F*) has been shown to remain stable during dry and rainy seasons [50].

The antibiotics present at sub-inhibitory concentrations in direct hospital effluents increase selection pressure, causing the cellular function of even normal microorganisms in the aquatic environment to change their genetic expression of virulence factors, or acquire resistance genes by different transfer mechanisms, posing a serious threat to public health, as suggested by Girijan et al. [13].

### 3.5. Composition of the Antibiotic-Resistant Bacterial Community and ARGs

Concerning the composition of the bacterial community and ARGs between the surface water samples and the sediment, differences are evidenced in several reviewed articles. In the evaluation of lakes in China, *α-Proteobacteria* were dominant in surface waters, and *Cyanobacteria*, in sediment samples. Similar results were obtained in relation to the types of ARGs, whereby the ARG profiles in surface waters were dominated by *strA* and *dfrE*, and in the sediments genes were found related to efflux pumps (*acrB, acrD, acrF, adeG*, *adeJ, armB, ceoB mdrB, mdrC, mexB, mexY, smeE*) [12].

Guan et al. [18] reported that the absolute abundance of ARGs (*tetC, bla*_TEM_*, ermF, sul1, cmlA* and *gyrA*) in sediments in some rivers in China was 1 to 3 orders of magnitude higher than in the surface water. In the bacterial community of sediments, the relative abundances of *Acidobacteria*, *Chloroflexi*, *Spirochaetes*, *Chlamydiae* and *Aminicenantes* were higher than 1%, while in surface water the percentage was less than 1%, sediments being an environment with greater diversity and bacterial richness. The specific resistance gene to bacitracin was very abundant in the sediments of the Pearl River in China, as reported by Chen et al. [69].

In the WWTPs, a similar behavior was observed, whereby the active sludge samples were dominated by nitrifying agents and *E coli* (8–11%), while on the surface *Pseudomonas* spp. (20–22%), *Mycobacterium* spp. (17–19%) and *Arthrobacter* sp. (13–14%) were more dominant [48]. 

**Figure 8:**
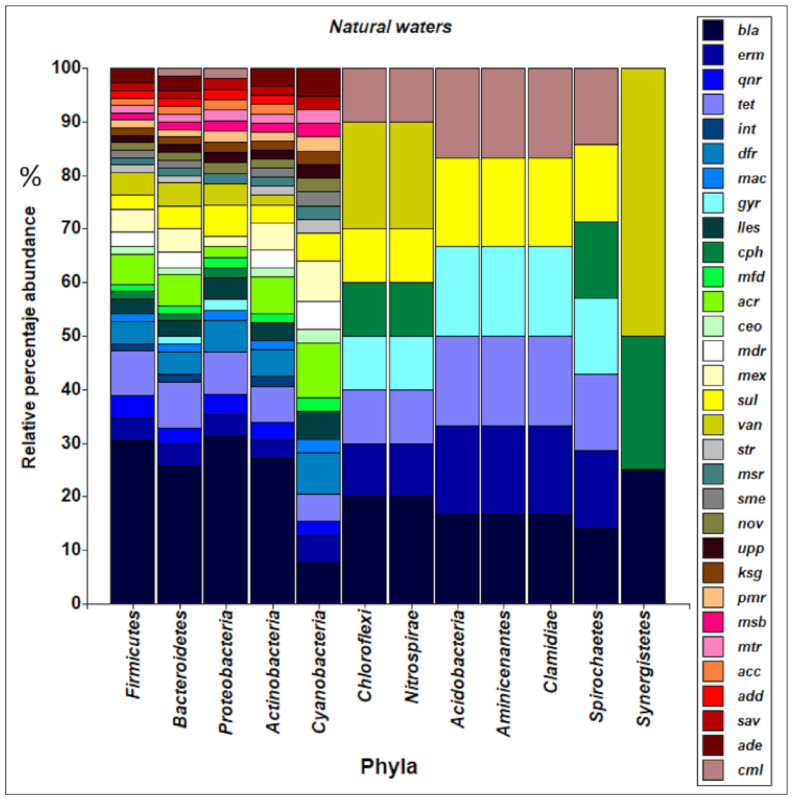
Abundance and diversity of ARG and bacterial taxa present in natural water. *bla* (gene with resistance to betalactamase), *erm* (gene with resistance to erythromycin), *qnr*(gene with resistance to quinolones), *tet* (gene with resistance to tetracycline), *int* (integron), *dfr* (gene with resistance to trimethoprim), *mac* (gene with resistance to macrolides), *gyr* (gene encoding a subunit of DNA gyrase), *iles* (gene with resistance to quinolones), *cph* (gene with resistance to imipenem), *mfd* (Mutation-Frequency-Decline: onfers resistance to the host nitrogen immune response), *acr (*gene that codes for acrosin), *ceo* (gene with resistance to cefoperazone), *mdr* (The multiple drug resistance gene *mdr* encodes the so-called P-Glycoprotein), *mex* (gene with resistance to tetracycline), *sul* (gene with resistance to Sulfamethazine), *sul* (gene with resistance to Sulfamethazine), *van (*gene with resistance to vancomicine), *str* (gene with resistance to streptomycin), *msr* (gene with resistance to macrolides, lincosamides and streptogramins), *sme (*gene with resistance to carbapenems), *nov (*gene with resistance to novobiocine) *upp* (Uracil fosforribosiltransferasa), *ksg* (Serine/threonine-protein kinase), *pmr* (gene with resistance to peptide antibiotic), *msb* (gene with resistance to nitroimidazole antibiotic), *mtr* (gene with resistance to penam, macrolide antibiotic), *acc* (gene with resistance to penam), *add*(gene with resistance to kanamycin), *sav* (efflux pump), *ade* (gene with resistance to tetracycline and glycylcycline), *cml* (gene with resistance to cloranphenicol).

The articles included in this review incorporate a database that could be robustly analyzed for patterns of resistance to antibiotics in bacterial communities in the aquatic environment. In natural waters, *bla* genes predominate in *α-Proteobacteria*, (31.4%), *Firmicutes* (30.4%), *Actinobacteria* (27.1%) and *Bacteroides* (25.7%). *tet* and *dfr* genes were distributed at between 8.6 and 6.8% and 4.2 and 5.9%, respectively, in these phyla (Figure 8).

**Figure 9:**
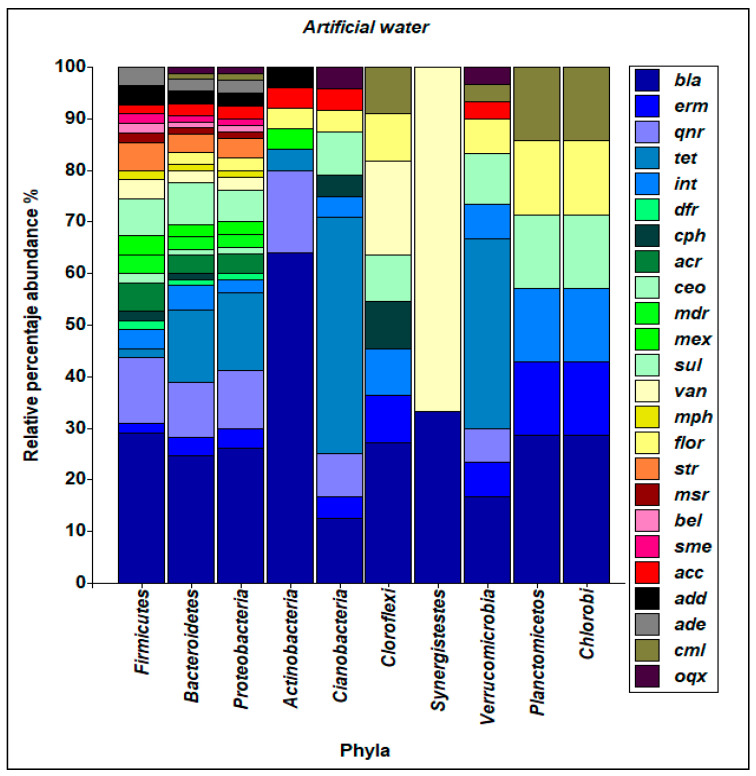
Abundance and diversity of ARG and bacterial taxa present in artificial waters. *bla* (gene with resistance to betalactamase), *erm* (gene with resistance to erythromycin), *qnr*(gene with resistance to quinolones), *tet* (gene with resistance to tetracycline), *int* (integron), *dfr* (gene with resistance to trimethoprim), *cph* (gene with resistance to imipenem), *acr (*gene that codes for acrosin), *ceo* (gene with resistance to cefoperazone), *mdr* (The multiple drug resistance gene *mdr* encodes the so-called P-Glycoprotein), *mex* (gene with resistance to tetracycline), *sul* (gene with resistance to Sulfamethazine), *sul* (gene with resistance to Sulfamethazine), *van (*gene with resistance to vancomicine), *mph (*gene with resistance to macrolides), *floR* (gene with resistance to fluoroquinlones), *str* (gene with resistance to streptomycin), *msr* (gene with resistance to macrolides, lincosamides and streptogramins), *bel* (type of extended spectrum beta-lactamase), *sme (*gene with resistance to carbapenems), *acc* (gene with resistance to penam), *add*(gene with resistance to kanamycin), *cml* (gene with resistance to cloranphenicol), *oqx* (gene with resistance to tetracycline).

A greater richness of the *bla* genes is evidenced in the phyla *Actinobacteria* (65.1%), *Firmicutes* (29.1%), *α-Proteobacteria* (25.9%) and *Bacteroides* (24.7%) in waters of artificial origin. The *dfr* genes were distributed in the range of 10.6 to 15.4% among these phyla. The *tet* genes were more frequently detected in the phyla *Firmicutes* (18%), *α-Proteobacteria* (14.8%), *Bacteroides* (14.1%), *Actinobacteria* (3.8%), *Cyanobacteria* *(*47.8%) and *Verrucomicrobia* (36.7%), as shown in Figure 9.

### 3.6. Mobile Genetic Element

The transfer of ARGs between bacteria in aquatic environments is facilitated through mobile genetic elements. The reviewed studies report the presence of plasmids, integrons and transposons, which corroborates that these are the most important mobile elements in the spread of ARGs in aquatic environments, as shown in Tables S2 and S3 (Supplementary Materials).

Several articles show a significant correlation between mobile genetic element and ARGs [20,22,30,31,36,51,55]. Fang et al. [51] suggested that plasmids were the most abundant elements and were strongly correlated with ARGs. In strains of *Aeromonas* spp., carriers of the *bla_KPC-2_* gene, obtained from a WWTP in Japan, the transfer of this gene was evidenced through *IncP-6* plasmids and I*S26* transposon, which includes a macrolide resistance gene (*mphA*) [30].

Analysis of the microbial community revealed potential host bacteria for ARG and integrons. The *int-1* gene, and various types of ARGs, especially *bla*, were abundant in the sediments of aquaculture farms. The researchers found significant correlations between *int-1* abundance and total ARG abundance in these sediments [22]. The analysis of the waters from a treatment plant and a river near the city of Xiamen, China detected the ARGs *bla*_KPC-1_, *floR, sul-1* and *ermB,* and *intI-1.* Chen et al. [55] found a close relationship between the *sul-1* and *int-1* genes in several rivers of China. These results are consistent with those reported by Obayiuwana et al. [36] on pharmaceutical wastewaters from cities in southwestern Nigeria, Africa. 

These reports support the idea that integrons, transposons and plasmids play an important role in the spread of ARG in aquatic environments impacted by human activity. However, a large number of European and North American studies do not report on these mobile genetic elements; this makes it difficult to determine the frequency with which the selection and spread of ARGs occurs in aquatic environments around the world [11,12,27,29,47,57,58].

### 3.7. Effect of Exposure to WWTP Effluent in Aquatic Ecosystems

According to the articles reviewed, several authors suggest that the diversity of anti-biotic-resistant bacteria in aquatic habitats can be influenced by anthropogenic contamination. In wastewater and in WWTP, bacteria are exposed to multiple antibiotics, these being released into treated water until it reaches natural water systems, facilitating the spread of antibiotic resistance [29,56,76]. The uncontrolled discharge of untreated municipal waste can contribute to an overall increase in the abundance and diversity of ARGs in the environment, including those that confer resistance to last-resort antibiotics [18].

Mi et al. [76] showed that the proper maintenance of distribution and storage systems in communities is essential to provide access to clean drinking water. The authors showed that the WWTPs were working properly in several major cities in European countries, as the after-treatment water did not contain *E. coli* or coliforms. However, once the water entered the distribution system, a decrease in chlorine concentration was observed with a concomitant increase in bacterial counts. Wang et al. [19], with a step-by-step analysis regarding the impact of WWTPs on three pollutants, demonstrated that wastewater treatment was ineffective in removing the determinants of antimicrobial resistance from wastewater, and suggested that the design of WWTPs must be improved to address threats from these pollutants.

The highest numbers of ARGs were observed in samples of hospital wastewater and in natural water under the plant, while the lowest number was determined in water samples upstream of the WWTPs [10]. It is likely that the increase in ARG downstream is attributable to the accumulation of genes present in the treated effluent discharged from the WWTP [19,37,39,42].

Another way to impact natural water systems is through aquaculture. The metagenomic analysis of Nakayama et al. [73] in the Cai Rang River, Vietnam, impacted by the activity of aquaculture, showed the predominance of *Proteobacteria*, *Actinobacteria* and *Bacteroidetes*, representing 64% of the total microbiota. The most representative genera were *Polynucleobacter*, *Variovorax* and *Limnohabitans*, representing more than 78.4%. Residues of sulfamethoxazole and sulfadimidine were widely detected, together with genes *sul-1*, *sul-2* and *bla*_CTX-M-_*1*, suggesting that these freshwater systems may have been contaminated by human activity.

There were substantial differences in composition between the bacterial communities of natural and artificial waters. The treated wastewater effluent and natural waters show a more diverse community, where taxa are more abundant. On the contrary, wastewater was dominated by *Firmicutes* (49.7%) and, to a lesser extent, by *Gammaproteobacteria* (24.3%) [14]. The bacteria found in wastewater, including potential pathogens, contribute to maintaining the river bed resistome, and biofilms appear as sensitive biosensors to the effect of wastewater contamination in surface waters.

The abundance of potentially pathogenic bacteria is evident in water systems. Ma et al. [33] reported that the highest number of sequence fragments identified corresponded to *P. aeruginosa* (34%), with ARGs related to efflux pumps (*mexF* and *HAE1*) in drinking water. Jara et al. [39] analyzed samples from different areas with human influence in Antarctica, specifically on the Fildes Peninsula and King George Island, and detected an abundance of *Pseudomonas* sp. with resistance to antibiotics.

*P. aeruginosa* is a notoriously difficult-to-treat pathogen that can cause serious illness and infection, and therefore the high frequency of *Pseudomonas*-borne ARGs in the aquatic environment may increase the risk of infection and the ineffectiveness of antibiotics in hu-mans. In the river water samples, *bla*_KPC_ was detected in strains of *K. pneumoniae* [16], as well as *sul-1* and *sul-2*, in Gram-negative enteric bacteria of clinical interest [20]. Eduardo-Correia et al. [57] suggested that one or a few bacterial members of the community may be important promoters of the spread of antibiotic resistance in the bacterial population of the environment.

The increasing anthropogenic and industrial activity near or directly in water systems has contributed to the growth and strengthening of the bacterial resistome [7,9,41,61]. There is no doubt that aquatic environments act as reservoirs for the acquisition and spread of bacteria with resistance to antibiotics, so human exposure to bacteria resistant to antibiotics and ARGs from these environments may represent an additional risk to health.

### 3.8. Limitations of the Study

Most of the studies are from Asia. This fact may introduce a bias in the information, due to having a greater quantity of data from a single region, although we take into account that the resistance to antibiotics of bacteria found in water systems is a global problem.

A bias in the results of this review may be related to the fact that most articles do not report the biochemical parameters that explain the characteristics of the aquatic environment, which are closely related to the composition and abundance of the bacterial community and the types of ARGs. Furthermore, several articles found in Latin America do not include metagenomic analysis from pyrosequencing or Illumina technology.

Of the articles included in this systematic review, not all reported the Shannon diversity index (H’), so it was not possible to perform a comparative analysis in relation to the diversity index of the phyla found.

## 4. Conclusions

The present systematic review provides an overview of current information related to bacterial communities with resistance to antibiotics and their dynamics in aquatic environments. The studies from river water samples and from WWTP effluents are the most representative, and help to establish the microbial dynamics present in natural and artificial water systems, respectively.

An interesting piece of information provided by this systematic review was the high number of studies using sequencing/pyrosequencing (28 articles included), which provided comprehensive data for several bacterial taxonomic levels (e.g., species, genus, phylum). In addition, this type of analysis may detect unclassified microorganisms or uncultured bacterial species that could be associated with resistance to antibiotics of major clinical importance. The articles that used this technique were principally published in China and countries in Europe and North America. However, in Latin American and African countries, there is evidence of the low production of articles of this type, perhaps due to the high costs required to use this technique.

The results of the included studies showed a low diversity in the microbial communities of natural and artificial waters. The most prevalent phyla are *Bacteroidetes* and *α-Proteobacteria*. These phyla contain a high number of bacterial genera related to anthropogenic activity. In addition, the *bla* genes predominated in these bacteria; this confers resistance to beta-lactam antibiotics, which are the most widely used in medical practice worldwide. Interestingly, in the aquatic environments of urban areas of China, the most prevalent ARGs are the *tet* genes, associated with the intensive use of tetracycline in China, especially in aquaculture. 

The predominance of Gram-negative bacteria of the *Enterobacteraceae* family with resistance to antibiotics’ transporting mobile elements, such as plasmids and integrons, in aquatic systems, and especially in drinking water and natural water systems, confirms that WWTPs are not capable of completely eliminating antibiotics, resistant bacteria and ARGs. Therefore, they are potential reservoirs of resistance to antibiotics.

The ARGs reported mainly were those that confer resistance to β-lactam antibiotics, aminoglycosides, fluoroquinolones, macrolides and tetracyclines. These antibiotics are the most widely used in clinical practice and, when eliminated through water systems, exert selective pressure on environmental bacteria, allowing the spread of resistance, which constitutes a problem for human and animal health.

In this review we found that some articles were limited to reporting only ARG types and frequency, and others were based on studies of cultured bacteria. This fact can make it difficult to understand the structure and diversity of bacterial communities in different aquatic environments, in relation to establishing the dynamics of these communities in the face of antibiotic resistance according to geographic location.

## Data Availability

Not applicable.

## Supplementary Materials

The following are available online at https://www.mdpi.com/1660-4601/18/5/2348/s1, Table S1: Raw Data, Table S2: Data Natural waters, Table S3: Data artificial waters, Figure S1: Articles x country.
